# Immunogenicity and vaccine potential of clinical isolate *Mycobacterium kansasii* strain against *Mycobacterium tuberculosis* infection

**DOI:** 10.1128/spectrum.00819-24

**Published:** 2024-07-09

**Authors:** Hongmin Kim, Sung Jae Shin

**Affiliations:** 1 Department of Microbiology, Institute for Immunology and Immunological Diseases, Brain Korea 21 PLUS Project for Medical Science, Yonsei University College of Medicine, Seoul, South Korea; City of Hope, Duarte, California, USA

**Keywords:** *Mycobacterium kansasii*, *Mycobacterium tuberculosis*, clinical isolate strain, Th1 response, tuberculosis vaccine

## Abstract

*Mycobacterium kansasii* is a bacterium included in non-tuberculous mycobacteria (NTM) that can cause lung disease. It shares a significant number of antigens with *Mycobacterium tuberculosis* (Mtb), suggesting that it has the potential to be used as a tuberculosis (TB) vaccine. Therefore, we subcutaneously vaccinated mice with reference strain, *M. kansasii*-ATCC12478 [*M. kansasii*-American Type Culture Collection (ATCC)], and clinically isolated strain, *M. kansasii*-SM-1 to evaluate potential as a TB vaccine by comparing with bacille Calmette-Guerin (BCG) vaccine. Ten weeks after vaccination, we evaluated immunogenicity of *M. kansasii*-ATCC and *M. kansasii*-SM-1, and *M. kansasii*-SM-1 immunization induces potent Mtb antigen-specific IFN-γ-producing CD4^+^ T cells than *M. kansasii*-ATCC. Upon Mtb infection, *M. kansasii*-SM-1 provided better protection than *M. kansasii*-ATCC, which was comparable to the efficacy of BCG. These results showed that the clinical strain *M. kansasii*-SM-1, which exhibits an enhanced Mtb antigen-specific Th1 response, shows greater vaccine efficacy compared to *M. kansasii*-ATCC. In this study, we demonstrated that vaccine efficacy can vary depending on the strain of *M. kansasii* and that its efficacy can be comparable to BCG. This suggests that *M. kansasii* has the potential to be a live TB vaccine candidate.

**IMPORTANCE:**

*Mycobacterium kansasii*, a non-tuberculous mycobacteria (NTM) species causing lung disease, shares key antigens with *Mycobacterium tuberculosis* (Mtb), indicating its potential for TB vaccine development. Subcutaneous vaccination of mice with *M. kansasii* strains reference strain *M. kansasii*-ATCC12478 [(*M. kansasii*-American Type Culture Collection (ATCC)] and clinically isolated strain *M. kansasii*-SM-1 revealed differences in immunogenicity. *M. kansasii*-SM-1 induced a robust Mtb antigen-specific IFN-γ-producing CD4^+^ T cell response compared to *M. kansasii*-ATCC. Additionally, *M. kansasii*-SM-1 conferred better protection against Mtb infection than *M. kansasii*-ATCC, which is comparable to bacille Calmette-Guerin (BCG). These findings underscore the variable vaccine efficacy among *M. kansasii* strains, with *M. kansasii*-SM-1 exhibiting promising potential as a live TB vaccine candidate, suggesting its comparative effectiveness to BCG.

## INTRODUCTION

Tuberculosis (TB) is a severe infectious disease caused by *Mycobacterium tuberculosis* (Mtb), and the Bacillus Calmette-Guérin (BCG) vaccine stands as the only licensed vaccine against TB for humans. While BCG effectively provides protective immunity in children, reducing the risk of Mtb infection progressing to active TB, its efficacy wanes in adults, particularly in cases of drug-resistant TB or when TB coincides with immune-compromising conditions like HIV infection or diabetes. Consequently, novel strategies are urgently required to overcome these limitations of BCG and enhance global TB control efforts.

Live attenuated vaccines are still favored for their capacity to elicit a wider immune response without necessitating adjuvants. According to recent studies, in pre-clinical stage, the positive results of vaccine candidates such as BCGΔBCG1419c (1) anda recombinant BCG that harbored the *esx-1* locus of *Mycobacterium marinum* (2) suggest the feasibility of developing a TB vaccine through a live vaccine. Moreover, VPM1002 and MTBVAC that have genetic background of mycobacteria are currently in phase 3 clinical trials (3).

Non-tuberculous mycobacteria (NTM) are mycobacterial species distinct from the Mtb complex and *Mycobacterium leprae*. Although generally perceived as less virulent than Mtb, NTM can lead to pulmonary and extrapulmonary diseases in susceptible individuals (4). In addition, NTM infection has been hypothesized to affect to vaccine-mediated protection and immunity against TB (5–7). These results may be because NTM shares a large number of antigens with BCG and Mtb and has cross activity with each other. Some NTM infection, such as *Mycobacterium kansasii* can induce positive results in the gamma interferon release assay (IGRA) for Mtb infection (8).

Early studies suggest that NTM infection provides animals with protection against Mtb infection (9–11). Recently, Podell et al. introduced a mouse model (BCG+NTM) to mimic BCG immunization combined with continuous NTM exposure, which exhibited enhanced and prolonged defense against pulmonary TB compared to BCG alone (12). Furthermore, regardless of BCG vaccination, high-dose NTM exposure provided partial protection against Mtb infection. Furthermore, Poyntz *et al.* further demonstrated that the administration of killed *Mycobacterium avium* enhanced the protective efficacy of BCG (13). These reports indicate the potential utility of NTM as a TB vaccine.

*M. kansasii* is primarily transmitted through the respiratory tract, and belongs to the NTM family, which consists of 170 species of mycobacteria. This bacterium is one of the major causative bacteria of human lung disease along with *M. avium* complex and *Mycobacteroides abscessus* (14). In a previous study, we characterized clinically isolated *M. kansasii* strains and observed that rapidly proliferating strains in bone marrow-derived macrophage increased TNF-α production and induced greater cell death (15). Previous literature has compared the apoptosis patterns of host cells using various BCG strains and suggested that induced apoptosis may provide enhanced protection against TB through the BCG vaccine (16).

Hence, our goal is to assess the immunogenicity and vaccine efficacy of two distinct strains, the reference strain *M. kansasii*-ATCC12478 [(hereafter *M. kansasii*-American Type Culture Collection (ATCC)] and clinically isolated strain *M. kansasii*-SM-1 in comparison to BCG against Mtb infection. We administered subcutaneous vaccinations of these two *M. kansasii* strains to C57BL/6 mice. The findings revealed that both strains of *M. kansasii* demonstrated a comparable level of immunogenicity to BCG. Moreover, it was confirmed that vaccination with *M. kansasii* provided equivalent or enhanced protection compared to BCG after 6 weeks against Mtb infection.

## RESULTS

### The comparison of the immunogenic response of *M. kansasii*-ATCC- and *M. kansasii-*SM-1-immunized mice

**FIG 1:**
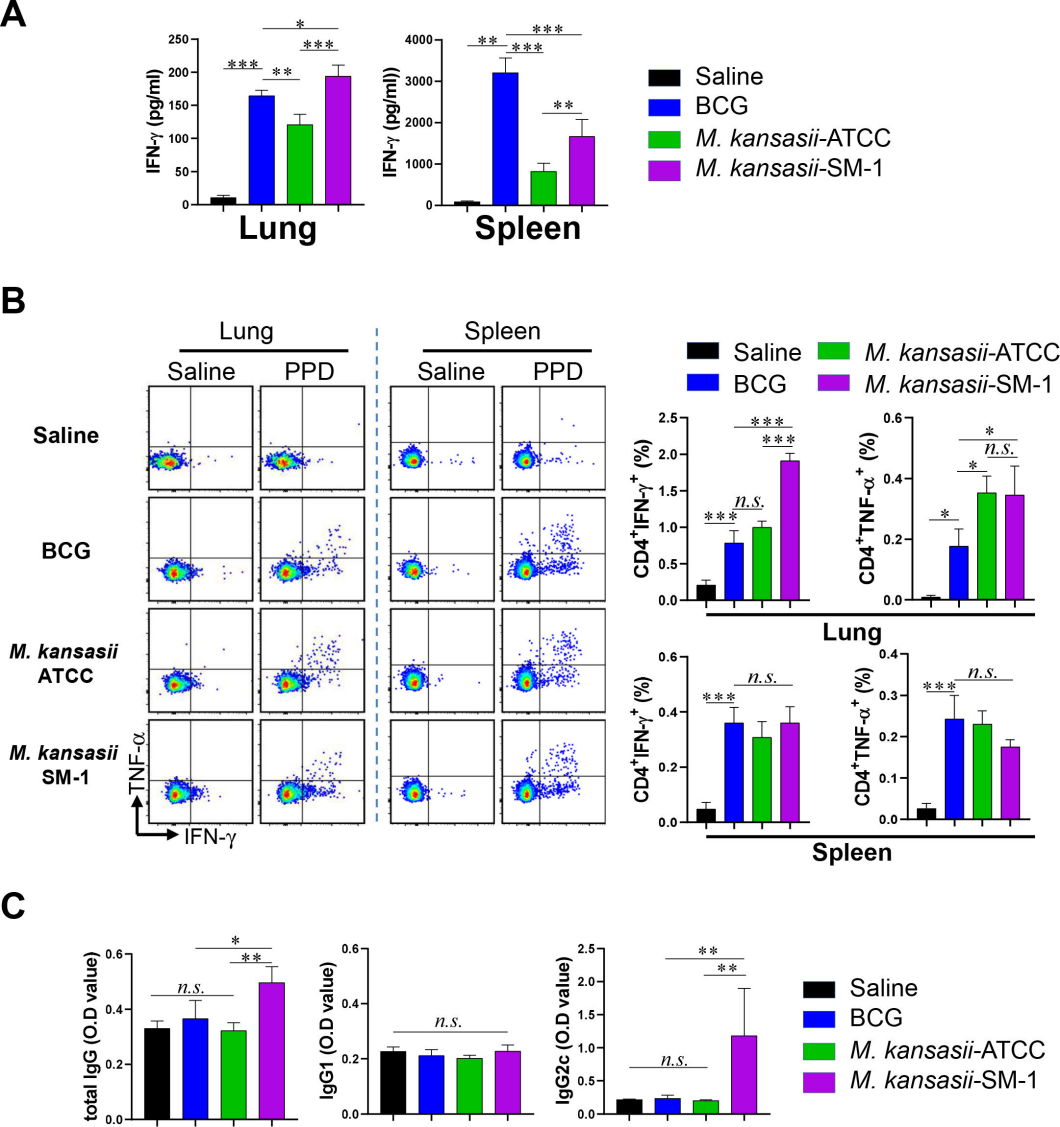
Immunogenicity of *M. kansasii-*ATCC- and *M. kansasii-*SM-1-immunized mice. Mice in each group (*n* = 5 mice/group) were vaccinated with either *M. kansasii*-ATCC, *M. kansasii*-SM-1, or BCG. After 10 weeks, their splenocytes and lung cells were cultured and stimulated with PPD (5 ug/mL) for 12 hours at 37°C. (A) The secretion of IFN-γ in response to PPD stimulation was assessed using ELISA. (B) Splenocytes and lung cells were stimulated with PPD in the presence of Golgiplug and GolgiStop for 12 hours at 37°C to analyze IFN-γ or TNF-α-producing CD4^+^ T cells via flow cytometry. Representative plots and bar graphs were generated to depict the levels of IFN-γ or TNF-α-producing CD4^+^ T cells in the lung and spleen of each vaccinated group. (C) The levels of PPD-specific IgG1, IgG2c, and total IgG in mouse serum were quantified using ELISA. Data are presented as means ± SDs from five mice in each group, and one-way ANOVA was employed to assess the significance of differences. *P < 0.033, **P < 0.002, ***P < 0.001; *n.s*., not significant.

IFN-γ and TNF-α are essential cytokines of CD4^+^ type 1T helper (Th1) cells, serving as markers recognized by the host immune system (17). Hence, we initially examined the Purified Protein Derivative (PPD)-specific IFN-γ production prior to Mtb strain challenge using both ELISA and flow cytometry. We immunized mice with *M. kansasii-*ATCC and *M. kansasii*-SM-1, or BCG subcutaneously. Ten weeks after the vaccination, BCG-, *M. kansasii-*ATCC-, and *M. kansasii-*SM-1-vaccinated groups were euthanized for analysis. The lungs and spleens were excised and processed to generate single-cell suspensions. Subsequently, these suspensions were exposed to 5 µg/mL of PPD for a duration of 12 h. Following incubation, the levels of IFN-γ secreted by the lung and spleen cells were quantified (Fig. 1A). Lung cells from every vaccinated group produced IFN-γ in response to PPD stimulation, but the *kansasii-*SM-1-immunized group was higher than that obtained with the *kansasii-*ATCC and BCG. Meanwhile, in spleen cells, the BCG vaccinated group released the most potent IFN-γ. In addition, IFN-γ- or TNF-α-producing CD4^+^ T cell populations in spleen and lung were analyzed in the immunized groups (Fig. 1B). Consequently, elevated frequencies of CD4^+^ T cells producing IFN-γ or TNF-α were detected in the lung, but no significant were detected in spleen. The populations of CD8^+^ T cells producing IFN-γ also exhibited similar patterns to those of CD4^+^ T cells in the lung, but the frequency was lower than that of CD4^+^ T cells (Fig. S1). The levels of PPD-specific immunoglobulin G (IgG), IgG2c, and total IgG were detected by ELISA. Although there was no statistical difference between the BCG and *M. kansasii-*ATCC groups, the *M. kansasii-*SM-1-immunized group showed higher level of IgG2C and total IgG than other groups (Fig. 1C).

### Comparison of Mtb antigen-specific multifunctional T cell responses in mice immunized with *M. kansasii*-ATCC and *M. kansasii*-SM-1 and mice immunized with BCG before Mtb challenge

**FIG 2:**
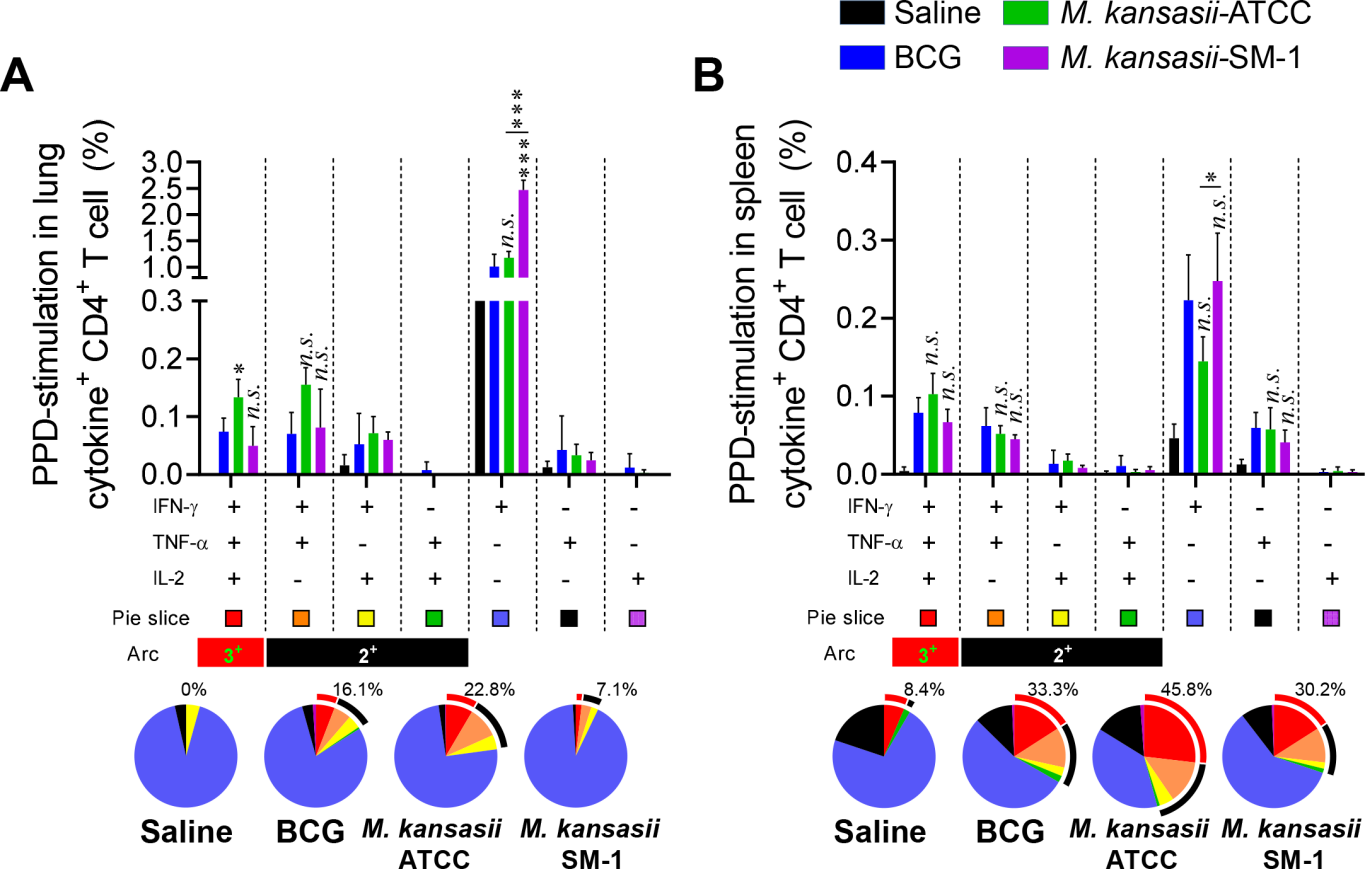
Comparison of the induction of Mtb antigen-specific multifunctional T cells in mice immunized with *M. kansasii-*ATCC and *M. kansasii-*SM-1. Ten weeks post-immunization, mice from each group were euthanized, and their lung and spleen cells were subjected to *ex vivo* stimulation with PPD (5 µg/mL) for 12 h at 37°C. Subsequently, the percentages of PPD-specific CD4^+^CD62L^lo^CD44^hi^ T cells producing IFN-γ, TNF-α, and/or IL-2 were analyzed among the isolated cells from both the (A) lungs and (B) spleens of each mouse group using flow cytometry. The frequencies and proportion of cells coexpressing IFN-γ, TNF-α, and/or IL-2 were represented in bar graph and pie charts. The arc around the pie chart indicates the proportion of T cells simultaneously producing more than two cytokines. Data are expressed as means ± SDs from five mice in each group, and statistical significance was determined using one-way ANOVA. *P < 0.033, **P < 0.002, ***P < 0.001; *n.s*., not significant.

Multifunctional Th1 cells, which produce IFN-γ, TNF-α, and/or IL-2, are considered crucial contributors to protection against TB in animal models, although the precise immune correlates of protection in humans remain elusive (3, 17–19). Thus, we next investigated the PPD-specific multifunctional T cell population. Individual live T cells were gated based on CD4^+^ or CD8^+^ expression, and triple-positive IFN-γ^+^TNF-α^+^IL-2^+^, double-positive IFN-γ^+^TNF-α^+^, IFN-γ^+^IL-2^+^, TNF-α^+^IL-2^+^, and single-positive IFN-γ^+^, TNF-α^+^, IL-2^+^ T cells were analyzed among the CD62L^lo^CD44^hi^ T cells (Fig. S2). Ten weeks after immunizations, the lung cells and splenocytes of the BCG-, *M. kansasii-*ATCC-, and *M. kansasii-*SM-1-immunized groups and the saline control group were restimulated with PPD. The groups immunized with the two strains of *M. kansasii* exhibited comparable frequencies of triple- and double-positive cytokine-secreting CD4^+^ T cells to those of the BCG-immunized group in both the lung and spleen, especially *M. kansasii-*SM-1-immunized group induce single IFN-γ producing CD4^+^ T cell in lung (Fig. 2A). However, among the spleen cells of all immunized groups, the frequency of cytokines-producing CD4^+^ T cells was not statistically different (Fig. 2B). CD8^+^ T cells showed an overall lower responsiveness to PPD stimulation compared to CD4^+^ T cells, and no clear differences were found between immunized groups, but the *M. kansasii*-SM-1-immunized group showed potent single IFN-γ producing CD8^+^ T cells in lung and spleen both (Fig. S3). These findings indicate that the profiles of T cell immunity elicited by the two strains of *M. kansasii* are different.

### Evaluation of the protective efficacy of immunization with *M. kansasii*-ATCC and *M. kansasii*-SM-1 against Mtb H37Rv infection

**FIG 3:**
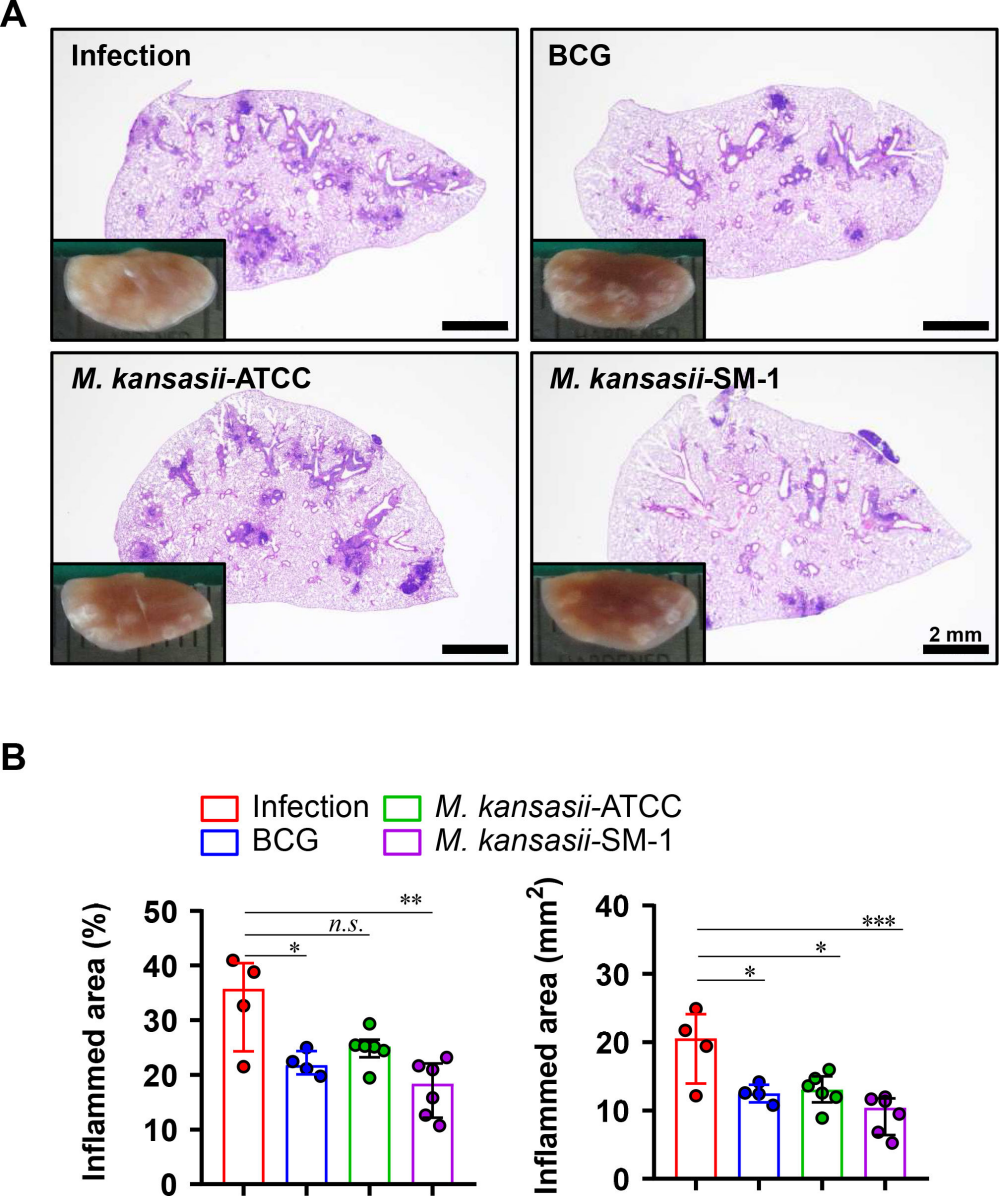
Histopathology of *M. kansasii-*ATCC- and *M. kansasii-*SM-1-immunized mice challenged with Mtb H37Rv. At 10 weeks post-immunization, the mice underwent challenge with 130 CFUs of the Mtb H37Rv strain via the aerosol route. (A) Subsequently, 6 weeks after challenge, the gross pathology of lung lesions was assessed through gross pathology examination and H&E staining of the superior lobe of the right lung. Representative changes in lung pathology among the various groups were documented. (B) Additionally, the percentage and size of the inflamed area in the superior right lung lobe were quantified and presented in dot graphs. Data are expressed as means ± SDs from four to six mice in each group, and statistical significance was determined using one-way ANOVA. *P < 0.033, **P < 0.002, ***P < 0.001.

After immunization, the Mtb strain H37Rv was challenged to mice to evaluate the protective efficacy of *M. kansasii-*ATCC and *M. kansasii-*SM-1 immunization. Mice were exposed to the Mtb H37Rv strain via aerosol route 10 weeks following immunization. Lung pathology and bacterial growth in both the lung and spleen were assessed 6 weeks after infection. At the same timepoint, the gross pathology and hematoxylin and eosin (H&E) staining of lung sections from all the groups were performed (Fig. 3A and B). The inflamed lesions from every immunized group exhibited milder symptoms compared to Mtb infection control group. Compared with the infection control group, *M. kansasii*-SM-1 immunization resulted in a reduction of lesions comparable to BCG, but *M. kansasii*-ATCC showed lower protective efficacy than *M. kansasii*-SM-1.

**FIG 4:**
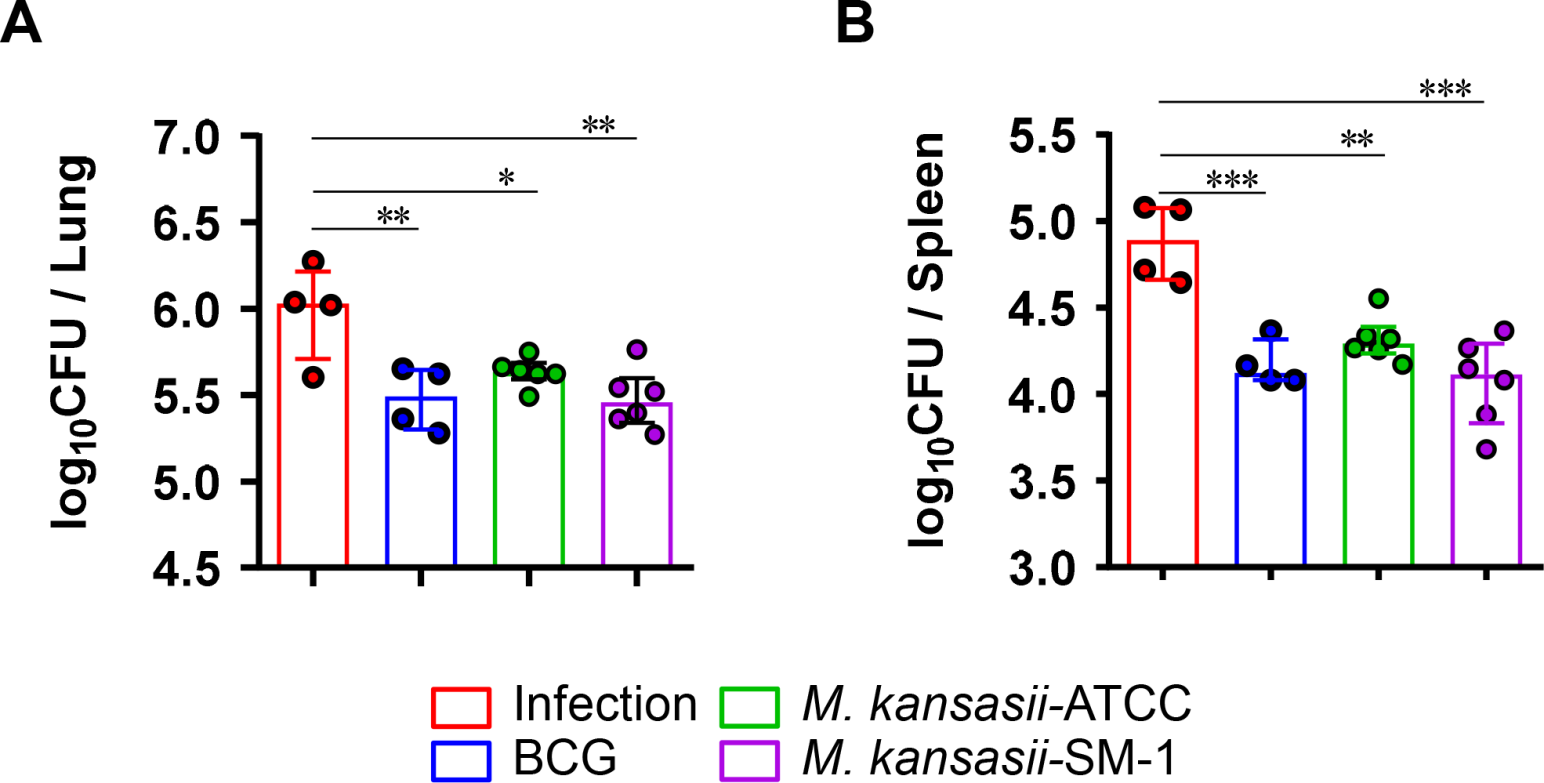
Bacterial burdens of *M. kansasii-*ATCC- and *M. kansasii-*SM-1-immunized mice challenged with Mtb H37Rv. The CFUs in the (A) lungs and (B) spleens of each group were analyzed by culturing lung and spleen homogenates and enumerating the bacteria. The data are presented as the medians ± interquartile range log_10_CFU/organ (*n* = 4–6), and the levels of the significance of the differences obtained in the comparisons among the samples were determined by one-way ANOVA followed by Dunnett’s test. *P < 0.033, ** *P* < 0.002, ***P < 0.001; *n.s*., not significant.

Similar to the results obtained regarding lesion appearance, controlled bacterial growth was observed in the lungs of all immunized groups compared to the infection control group. Compared with the infection control group, *M. kansasii*-SM-1 immunization resulted in a reduction of bacterial burden comparable to BCG in lung, but *M. kansasii*-ATCC showed lower protective efficacy than *M. kansasii*-SM-1 (Fig. 4A), and the same pattern was also observed in the bacterial burden of spleen (Fig. 4B). These results suggest that the different protective efficacy against Mtb infection may be dependent on the differences in *M. kansasii* strains, and that the clinical isolate *M. kansasii*-SM-1 may provide comparable protective efficacy to BCG.

### *M. kansasii* immunization induces Mtb antigen-specific multifunctional Th1 responses of spleen after Mtb H37Rv challenge

**FIG 5:**
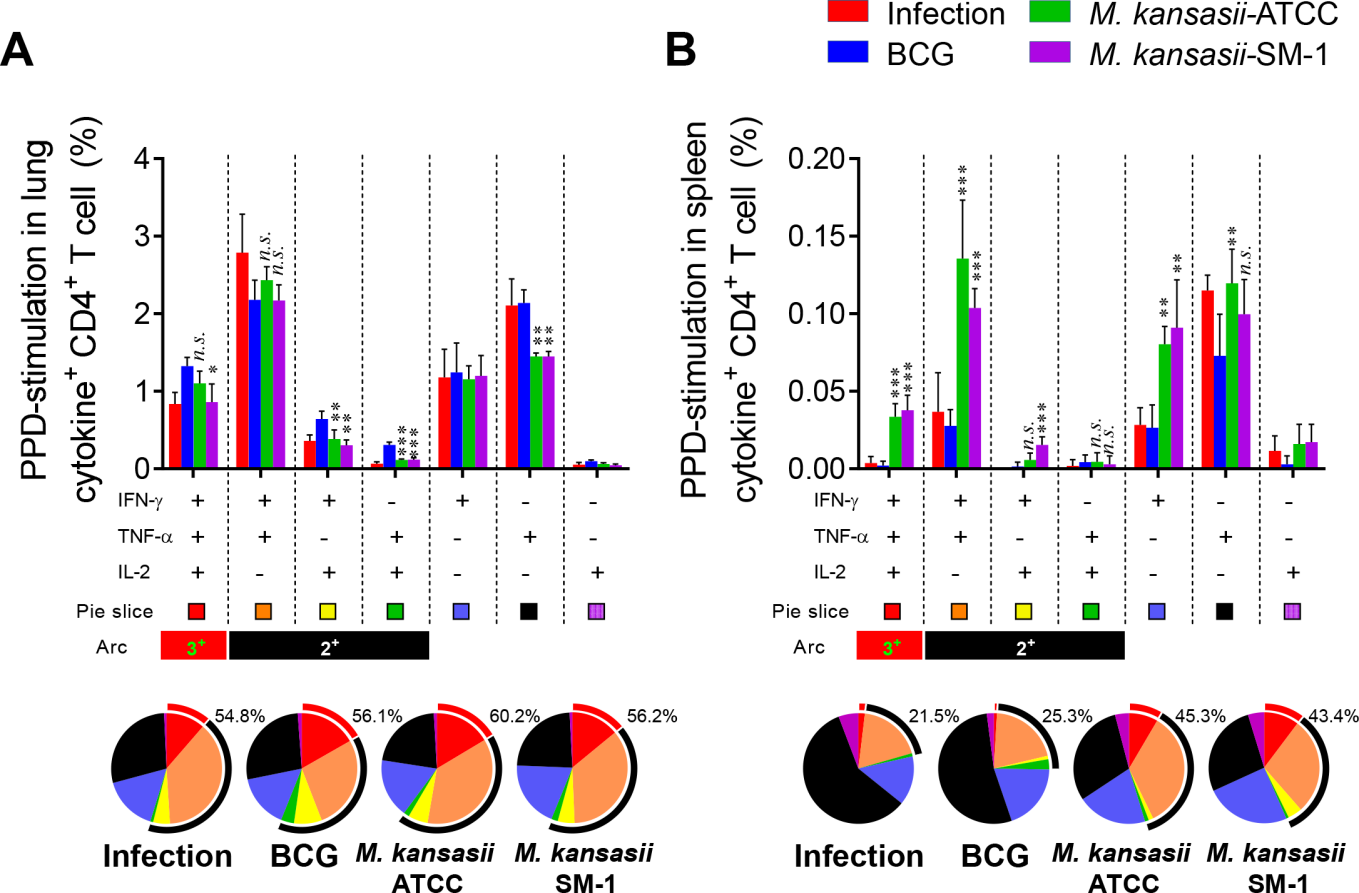
Mtb antigen-specific multifunctional T cell responses in *M. kansasii-*ATCC and *M. kansasii-*SM-1-immunized mice on Mtb H37Rv infection. Six weeks post H37Rv challenge, mice from each group were euthanized, and their lung and spleen cells were subjected to *ex vivo* stimulation with PPD (5 µg/mL) for 12 hours at 37°C. The percentages of PPD-specific CD4^+^CD62L^lo^CD44^hi^ T cells producing IFN-γ, TNF-α, and/or IL-2 cells from the (A) lungs and (B) spleens of each mouse group were analyzed using flow cytometry. The frequencies and proportion of cells coexpressing IFN-γ, TNF-α, and/or IL-2 were represented in bar graph and pie charts. The arc around the pie chart indicates the proportion of T cells simultaneously producing more than two cytokines. Data are presented as means ± SDs from four to six mice in each group, and statistical significance was determined using one-way ANOVA. *P < 0.033, **P < 0.002, ***P < 0.001; *n.s*., not significant.

Taking into account the immunization results, we further assessed whether immunization with *M. kansasii*-ATCC and *M. kansasii*-SM-1 maintains a protective T cell response upon challenge with H37Rv in both the spleen and lungs. For this analysis, Mtb infection control group and all immunized groups were sacrificed 6 weeks post-infection. Single cells from the lungs and spleens were subsequently stimulated with the immunogenic proteins PPD. In lung tissue, the frequency and proportion of CD4^+^ multifunctional T cells were similar in all groups regardless of immunization (Fig. 5A). Although there was no significant statistical difference between the immunization groups, the production of IFN-γ in lung cells by PPD stimulation showed the highest in the infection control group, probably correlated with CFU and lung pathology severity (Fig. S4). In contrast, there was no difference in PPD-induced production of IFN-γ in spleen cells between all groups (Fig. S4). However, the markedly higher frequency of IFN-γ^+^TNF-α^+^IL-2^+^, IFN-γ^+^TNF-α^+^, and IFN-γ^+^ producing-CD4^+^ T cell population was shown in spleen cells of *M. kansasii-*ATCC- and *M. kansasii-*SM-1-immunized groups. The proportion of multifunctional CD4 ^+^ T cells in *M. kansasii-*ATCC- or *M. kansasii-*SM-1-immunized groups were also higher than BCG-immunized group (Fig. 5B). In contrast, regarding the CD8^+^ T cell population, there was little difference in the frequency of the multifunctional T cell population among the *M. kansasii-*ATCC-, *M. kansasii-*SM-1-, and BCG-immunized groups in the lung and spleen, but rather higher levels of IFN-γ^+^TNF-α^+^ and TNF-α^+^ populations were detected in lung cells of infection control group (Fig. S5). Collectively, the findings indicate that immunization with *M. kansasii* not only induces CD4^+^ multifunctional T cell responses similar to BCG in the lung even after infection with Mtb but can also generate multifunctional Th1 CD4^+^ T cells that outperform BCG in the spleen.

## DISCUSSION

There have been studies on TB vaccines using various mycobacteria (20, 21). Early attempts to improve the protective efficacy of BCG by heterologously expressing ESX-1 from Mtb produced improved vaccine efficacy (22), but as a side effect, virulence was increased (23). In contrast, BCG expressing ESX-1 from *Mycobacterium marinum* showed lower virulence and improved vaccine efficacy (22). Recombinant BCG expressing the nontoxic mutant of *Escherichia coli* heat labile enterotoxin as adjuvant induces increased early and long-term immune responses against Mtb infection (2). However, in the case of NTM, the focus is mainly on the efficacy of BCG due to NTM exposure, and its potential as a TB vaccine has not been objectively evaluated. To date, it has been reported that NTM are an opportunistic pathogen that inhibits the efficacy of BCG and ultimately its protective effect against Mtb infection (24). However, several studies have reported that exposure to NTM increases the efficacy of BCG, or that NTM infection itself increases protection against Mtb infection (9–13).

In a previous our study, *M. kansasii*-SM-1 infection prompted elevated TNF-α production compared to *M. kansasii*-ATCC in bone marrow-derived macrophages (15). Additionally, the *M. kansasii*-SM-1 exhibited a higher growth rate compared to the *M. kansasii*-ATCC. Given that replication of BCG in vaccine recipients is necessary for eliciting and sustaining protective immunity (25, 26), these findings imply that *M. kansasii-*SM-1 may serve as a superior protection compared *to M. kansasii*-ATCC following immunization. Therefore, we investigated the immunogenicity and efficacy as a TB vaccine through immunization of *M. kansasii-*ATCC and the clinical isolate *M. kansasii-*SM-1.

In our research results, immunization with *M. kansasii*-SM-1 induced higher IFN-γ release and IFN-γ producing T cells response to PPD stimulation compared to BCG. These results indicate that immunization with *M. kansasii*-SM-1 can recognize Mtb antigens better than BCG immunization, suggesting an effective vaccine potential against Mtb infection. Some NTMs, such as *M. kansasii* and *M. marium*, have been reported to have *esat-6* and *cfp-10*, which are absent in most strains of NTM and BCG (27, 28). In addition, *M. kansasii* conserves virulence factors present in Mtb, such as the ESX-1 secretion system, PhoPR two-component system, and DosR/S/T regulon (29–31), but the presence of these antigens does not provide *M. kansasii* with the pathogenic capabilities of Mtb. Since these antigens are major antigens of Mtb that cause immunogenicity, the presence or absence of their expression in *M. kansasii* and BCG may be responsible for the differences in the IFN-γ response to PPD and protection against Mtb infection. Especially, the frequency of IFN-γ-producing Th1 response was significantly increased in *M. kansasii*-SM-1-immunized group compared to *M. kansasii*-ATCC before Mtb infection, and similar patterns were seen in the titer of PPD-specific IgG2c, which is an indirect measure of Th1 response. This difference may be due to the amount of antigen expression between *M. kansasii*-ATCC and *M. kansasii*-SM-1. For example, in our previous study, in the case of Mtb, we reported differences in the expression of key immunogenic antigens, such as ESAT-6 and CFP-10, between the H37Rv reference strain and the K strain, a clinical isolate belonging to the Beijing group (32). Additionally, Arend et al. reported differences in ESAT-6 and CFP-10 expression in 36 *M*. *kansasii* strains collected from both hospital and natural environments (28).

Previous studies reported that NTM can induce protection against Mtb infection (9, 11, 12). However, these reports were mainly conducted focusing on the effect of NTM on BCG vaccine efficacy. Therefore, because the route, inoculation dose, and timing of BCG and NTM vaccination were different, direct comparison of protective efficacy to Mtb infection was difficult. In addition, these results were due to aerosol infection or inoculation of NTM through the oral or IV route, but *M. kansasii* inoculation through the subcutaneous route did not affect the protection against Mtb infection (10, 33), which contradicts the results of our study. This discrepancy may be caused from differences in the method of subcutaneous vaccination. Previous studies using subcutaneous inoculation of *M. kansasii* were conducted through inoculation into the footpad area or tail base area (10, 33). In this study, *M. kansasii* was vaccinated into the loose skin over the neck, and BCG vaccination was performed using the same dose and route as the *M. kansasii* vaccination, objectively proving the potential of *M. kansasii* as a TB vaccine.

One of the limitations of our study is that we did not perform safety testing on the two strains of *M. kansasii* immunization. *M. kansasii* strains possess the virulence factor antigen of Mtb, but are not as virulent as Mtb. Regarding this, previous studies evaluating the virulence of *M. kansasii* show heterogeneous virulence in clinical isolates (34). Therefore, in order to evaluate the safety, clinical isolate *M. kansasii*-SM-1 should be used in animal models such as appropriate immunocompetent animal model.

Our study evaluated the immunogenicity and TB vaccine efficacy of *M. kansasii* through objective comparison with BCG through immunization of the main strain of *M. kansasii*, and the results demonstrated that it had similar levels of immunogenicity and vaccine efficacy as BCG. These results suggest that *M. kansasii* could also become the backbone of a new live TB vaccine candidate through vaccine strategy such as recombination or BCG-prime NTM-boost regimen.

## MATERIALS AND METHODS

### Mycobacteria strains preparation

All mycobacterial strains employed in this study were cultured and prepared according to previously outlined methods (15, 35): The *M. kansasii* reference strain (ATCC12478) was acquired from the ATCC located in Rockville, MD. and *M. kansasii-*SM-1 were isolated from individuals with NTM pulmonary diseases. The BCG vaccine strain (Pasteur 1173P2) was sourced from the Pasteur Institute in Paris, France. The Mtb H37Rv strain (ATCC 27294) was obtained from the ATCC in Manassas, VA.

### Immunization and challenge protocol

Mice received vaccination with either BCG or *M. kansasii* strain via subcutaneous injection, with a dose of 1 × 10^6^ CFUs per mouse. Ten weeks post-vaccination, mice were exposed to Mtb strain H37Rv via aerosol inhalation, following established protocols (35). Briefly, an airborne infection apparatus equipped with a calibrated inhalation chamber (Glas-Col, Terre Haute, IN, USA) was employed to administer approximately 130 viable bacteria per mouse.

### Antibodies and reagents

A LIVE/DEAD Fixable Near-IR Dead Cell Stain Kit was procured from Molecular Probes (Carlsbad, CA, USA). For flow cytometry analyses, the following antibodies were utilized: phycoerythrin (PE)-conjugated monoclonal antibody (mAb) against IFN-γ, allophycocyanin (APC)-conjugated mAb against TNF-α, violet 450-conjugated mAb against CD44, brilliant violet (BV) 605-conjugated mAb against Thy1.2, and PerCP-Cy5.5-conjugated mAb against CD4 were acquired from BD Bioscience (San Jose, CA, USA). Additionally, Alexa Fluor 700-conjugated mAb against CD62L and PE-Cy7-conjugated mAb against IL-2 were obtained from eBioscience (San Diego, CA, USA). BV 785 conjugated- mAb against CD8a was purchased from BioLegend (USA).

### Cytokine measurement

Single cells isolated from the lungs and spleens of mice infected with Mtb or immunized with Mtb were stimulated with PPD for 12 h at 37°C. Subsequently, the levels of secreted IFN-γ and TNF-α in the culture supernatant were quantified using a commercial ELISA kit, following the manufacturer’s instructions (BD Bioscience, San Jose, CA, USA).

### Ab titer measurement in serum

The levels of PPD-specific total IgG, IgG1, and IgG2c in serum were assessed to evaluate Mtb antigen-specific type 1 or type 2 immune responses. In brief, 96-well plates were coated with 2 µg/mL of PPD. After incubation with diluted serum, horseradish peroxidase (HRP)-conjugated antibodies against total IgG, IgG1 (BD Bioscience, San Diego, CA, USA), or IgG2c (Southern Biotech, Birmingham, AL, USA) were used as secondary antibodies. The optical densities (OD) were determined at 450 nm.

### Intracellular cytokine staining

Lung and spleen cells obtained from both immunized and Mtb-infected mice were stimulated with 5 µg/mL PPD at 37°C for 12 h in the presence of GolgiPlug and GolgiStop (BD Bioscience). Initially, the cells were washed with PBS, and the Fc receptors were blocked with anti-CD16/32 blocking Abs at 4°C for 15 min. Surface molecules were then stained using fluorochrome-conjugated antibodies against Thy1.2, CD4, CD8, CD44, and CD62L, along with the LIVE/DEAD Fixable Dead Cell Kit, for 30 min at 4°C. Following another PBS wash, the cells were fixed and permeabilized using Cytofix/Cytoperm (BD Biosciences) for 30 min at 4°C. After two washes with Perm/Wash (BD Biosciences), the permeabilized cells were stained with PE-conjugated anti-IFN-γ, APC-conjugated anti-TNF-α, and PE-Cy7-conjugated anti-IL-2 antibodies for 30 min at 4°C. Finally, the cells were washed twice with Perm/Wash and fixed with IC fixation buffer (eBioscience) for subsequent flow cytometry analysis.

### Analysis of histopathology and Mtb burden

The protective vaccine efficacy was determined through an analysis of the histopathology and bacterial growth in the lung and spleen. The organs were removed at 6 weeks after infection to determine the degree of protection at these time points. For the lung histopathology analysis, the right-superior lobes were preserved overnight in 10% formalin and embedded in paraffin. The lung was sectioned at 4–5  µm and stained with H&E. For the bacterial growth analysis, the lung and spleen were homogenized, and serially diluted samples were plated onto Middlebrook 7H11 agar plates (Becton Dickinson, Franklin Lakes, NJ, USA) supplemented with 10% OADC (Difco Laboratories), 2 µg/mL 2-thiophenecarboxylic acid hydrazide (Sigma-Aldrich, St. Louis, MO, USA) and amphotericin B (Sigma-Aldrich). After incubation at 37°C for 3–4 weeks, the bacterial colonies were counted.

### Statistical analyses

Statistical analyses were performed using GraphPad Prism V8.0 (GraphPad Software, San Diego, CA, USA). Differences between two groups were assessed using an unpaired Student’s *t*-test, while one-way ANOVA followed by Tukey’s multiple comparison tests was applied for data analysis involving more than two groups. The means (± standard deviations, SDs) represent all values. Statistical significance was considered at *P < 0.033, **P < 0.002, or ***P < 0.001.

## Data Availability

The data that support the findings of this study are available in the article and the Supplementary file 1.

## SUPPLEMENTAL MATERIAL

Supplemental material spectrum.00819-24-s0001.docx Fig. S1 to S5.

ASM does not own the copyrights to Supplemental Material that may be linked to, or accessed through, an article. The authors have granted ASM a non-exclusive, world-wide license to publish the Supplemental Material files. Please contact the corresponding author directly for reuse.
