# Supplementary material for: Immunogenicity and vaccine potential of clinical isolate Mycobacterium kansasii strain against Mycobacterium tuberculosis infection
Source: Microbiol Spectr. 2024 Jul 9;12(8):e00819-24. doi: 10.1128/spectrum.00819-24 (PMC11302008; doi:10.1128/spectrum.00819-24)
Supplement: Supplemental material — Fig. S1 to S5. [file spectrum.00819-24-s0001.docx]

**Supplementary Materials**

**Immunogenicity and vaccine potential of clinical isolate *Mycobacterium kansasii* strain against *Mycobacterium tuberculosis* infection**

Hongmin Kim*, Sung Jae Shin*

Department of Microbiology, Institute for Immunology and Immunological Diseases,

Brain Korea 21 PLUS Project for Medical Science,

Yonsei University College of Medicine,

Seoul 03722, South Korea

* Correspondence:

Sung Jae Shin, Department of Microbiology, Yonsei University College of Medicine, Seoul 03722, Republic of Korea; Email: sjshin@yuhs.ac

Hongmin Kim, Department of Microbiology, Yonsei University College of Medicine, Seoul 003722, Republic of Korea; Email: goldhm@yuhs.ac

**
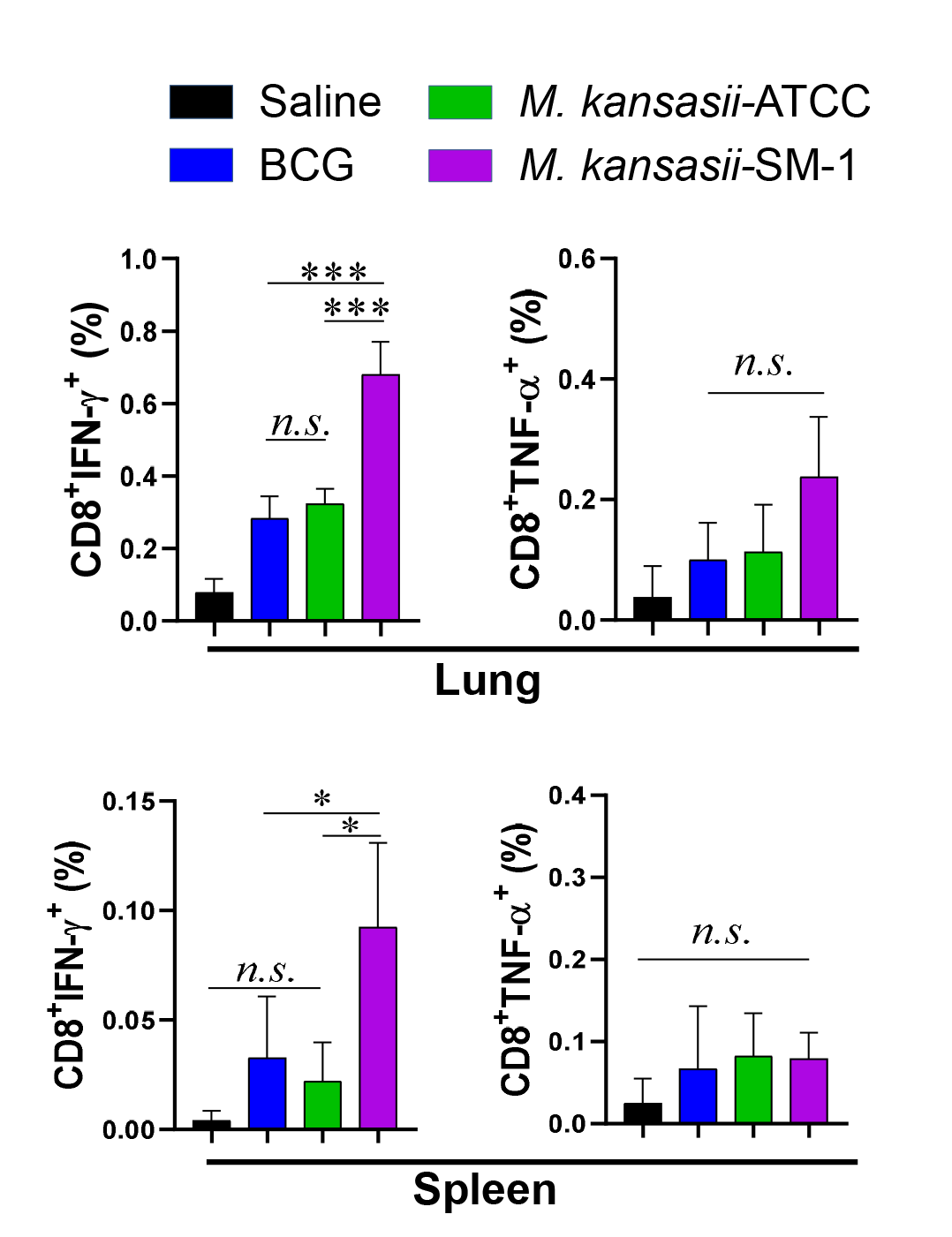
**

**Supplementary Figure 1. Analysis of PPD-specific CD8^+^ T cell responses**

Mice (n = 5 mice/group) were immunized with *M. kansasii-*ATCC, *M. kansasii-*SM-1, or BCG. At 10 weeks after the immunization, their lung and spleen cells were stimulated with PPD in the presence of Golgiplug and GolgiStop for 12 h at 37°C. IFN-γ or TNF-α-producing CD8^+^ T cells of spleen and lung were analyzed by flow cytometry. The representative plot and bar graph for IFN- γ- or TNF-α-producing CD8^+^T cells in every vaccinated group of lung and spleen.

**
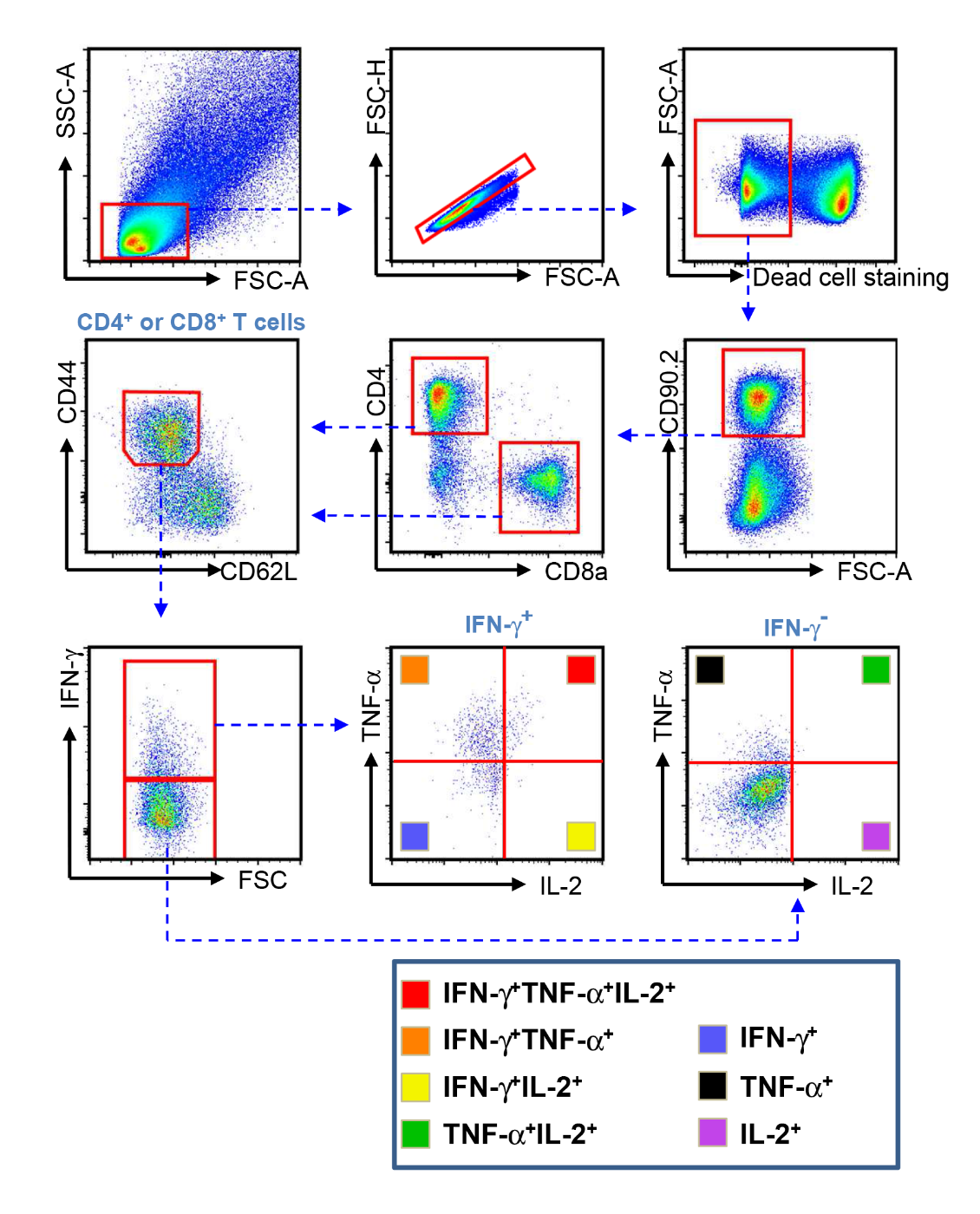
**

**Supplementary Figure 2.** **Gating strategy for the analysis of multifunctional T cells in the lungs and spleens**

The lymphocyte population was first gated based on their characteristic forward scatter (FSC) and side scatter (SSC) patterns. Single cells were gated based on equivalent FSC height (FSC-H) and FSC area (FSC-A) values to exclude doublets and large cell aggregates. For the analysis of living lymphocytes, the single cell-gated lymphocytes were stained with live and dead stains and Thy1.2 and then gated into CD4^+^ or CD8^+^ T cells. CD62L^lo^CD44^hi^ cells were gated to profile the T cells producing various cytokines (combinations of IFN-γ, TNF-α, and IL-2).


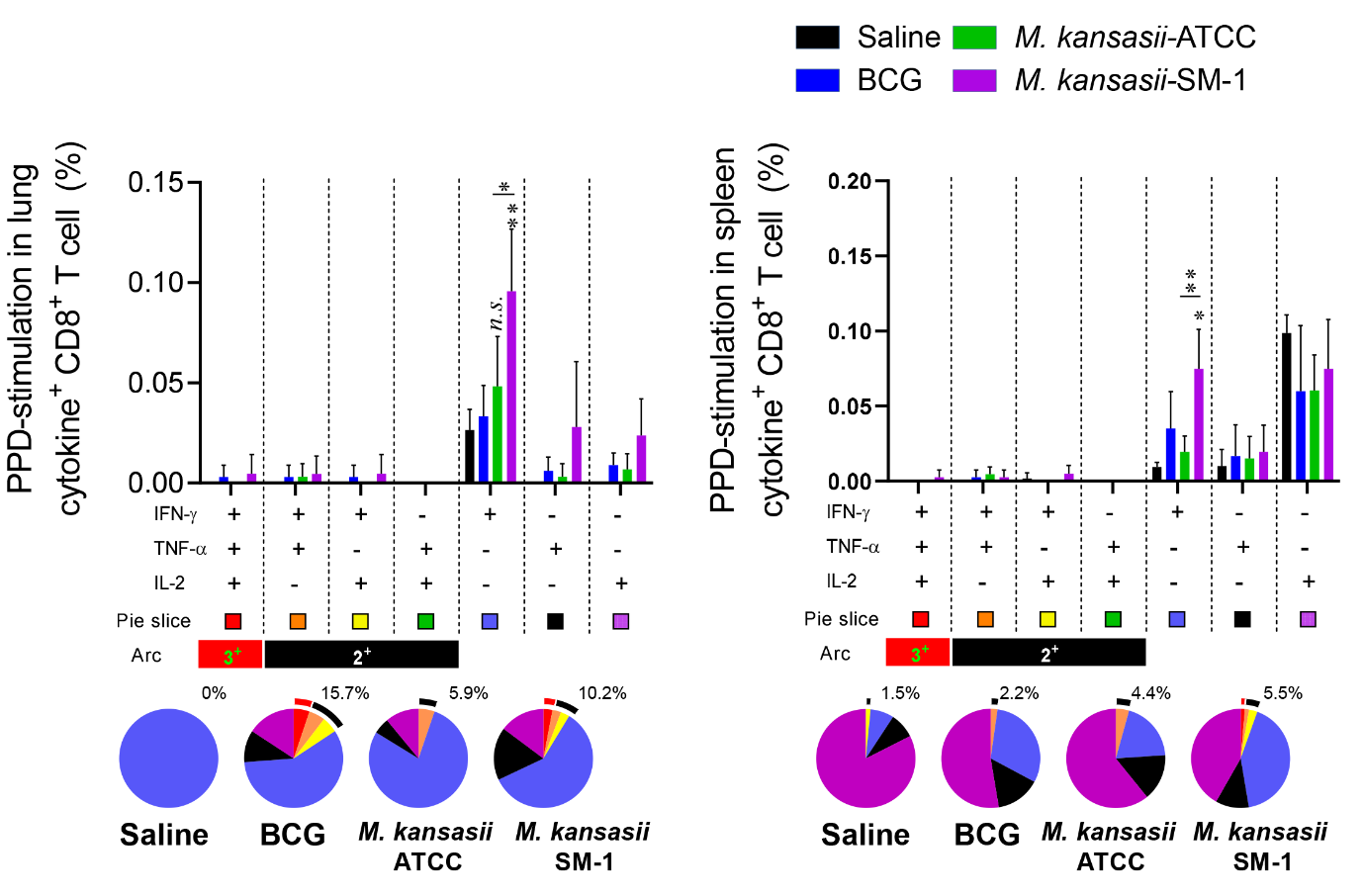


**Supplementary Figure 3. Analysis of Mtb antigen-specific CD8^+^ multifunctional T-cells in *M. Kansasii*-immunized mice**

Mice (n = 5 mice/group) were immunized with *M. kansasii-*ATCC, *M. kansasii-*SM-1, or BCG. At 10 weeks after the immunization, their lung and spleen cells were stimulated with PPD (5 μg/ml) in the presence of Golgiplug and GolgiStop for 12 h at 37°C. The percentages of PPD-specific CD8^+^CD62L^lo^CD44^hi^ T cells producing IFN-γ, TNF-α, and/or IL-2 among the cells isolated from the lungs and spleen of each group of mice were analyzed via flow cytometry. The frequencies and proportion of cells coexpressing IFN-γ, TNF-α, and/or IL-2 were represented in bar graph and pie charts. The arc around the pie chart indicates the proportion of T cells simultaneously producing more than two cytokines. The data are presented as the means ± SDs from five mice in each group. One-way ANOVA was used to determine the significance of the differences. **p* < 0.033, ** *p <* 0.002, *** *p <* 0.001; *n.s.*, not significant.


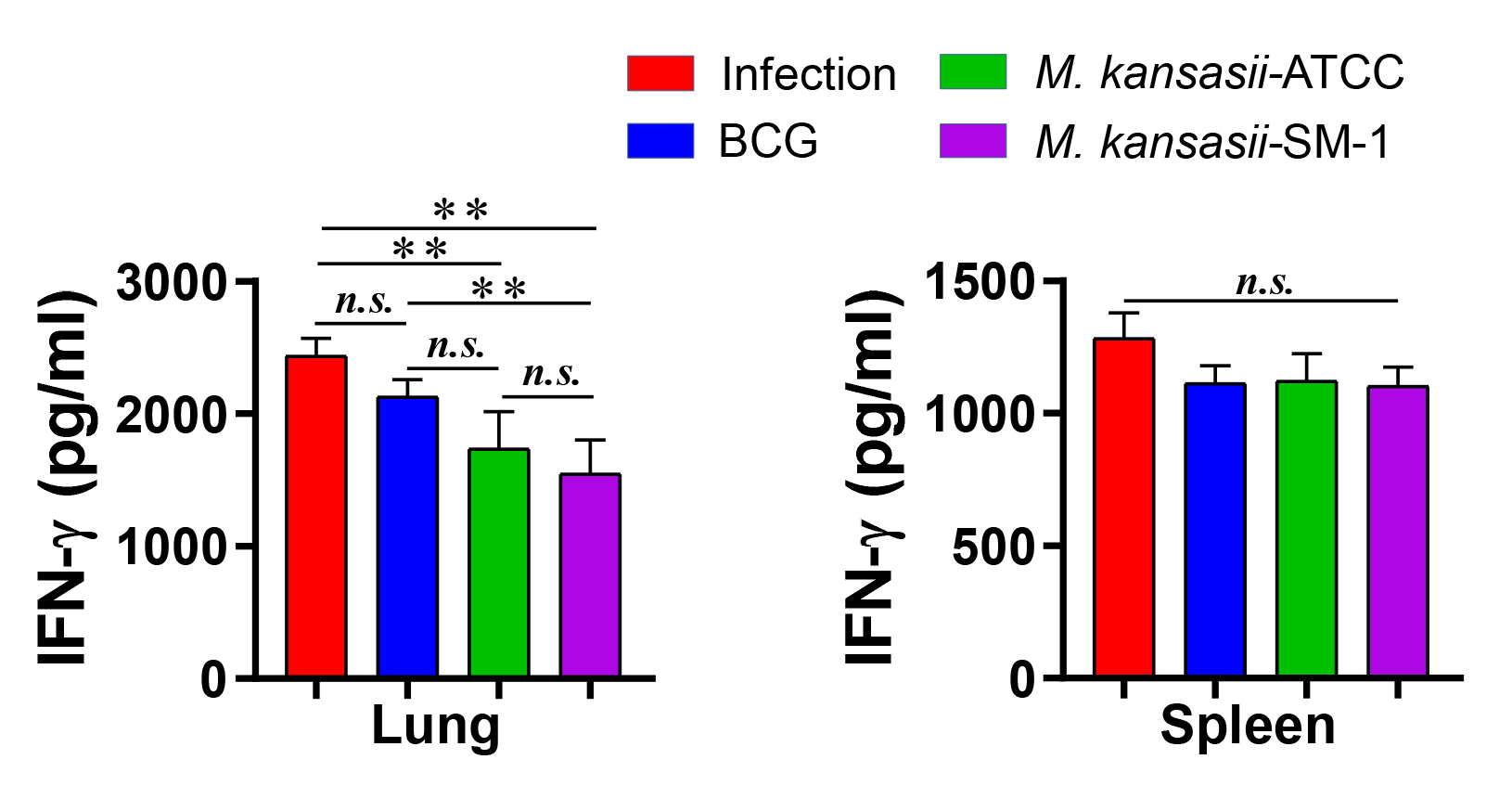


**Supplementary Figure 4. Measurement of IFN-γ secretion in response to PPD stimulation in lung and spleen cells**

Six weeks after the H37Rv challenge, the mice from each group were sacrificed, and their lung and spleen cells were stimulated ex vivo with PPD (5 μg/ml) for 12 hours at 37°C. The production of IFN-γ were measured by ELISA. The data are presented as the means ± SDs from four to six mice in each group. One-way ANOVA was used to determine the significance of the differences. **p* < 0.033, ** *p <* 0.002, *** *p <* 0.001; *n.s.*, not significant.

**
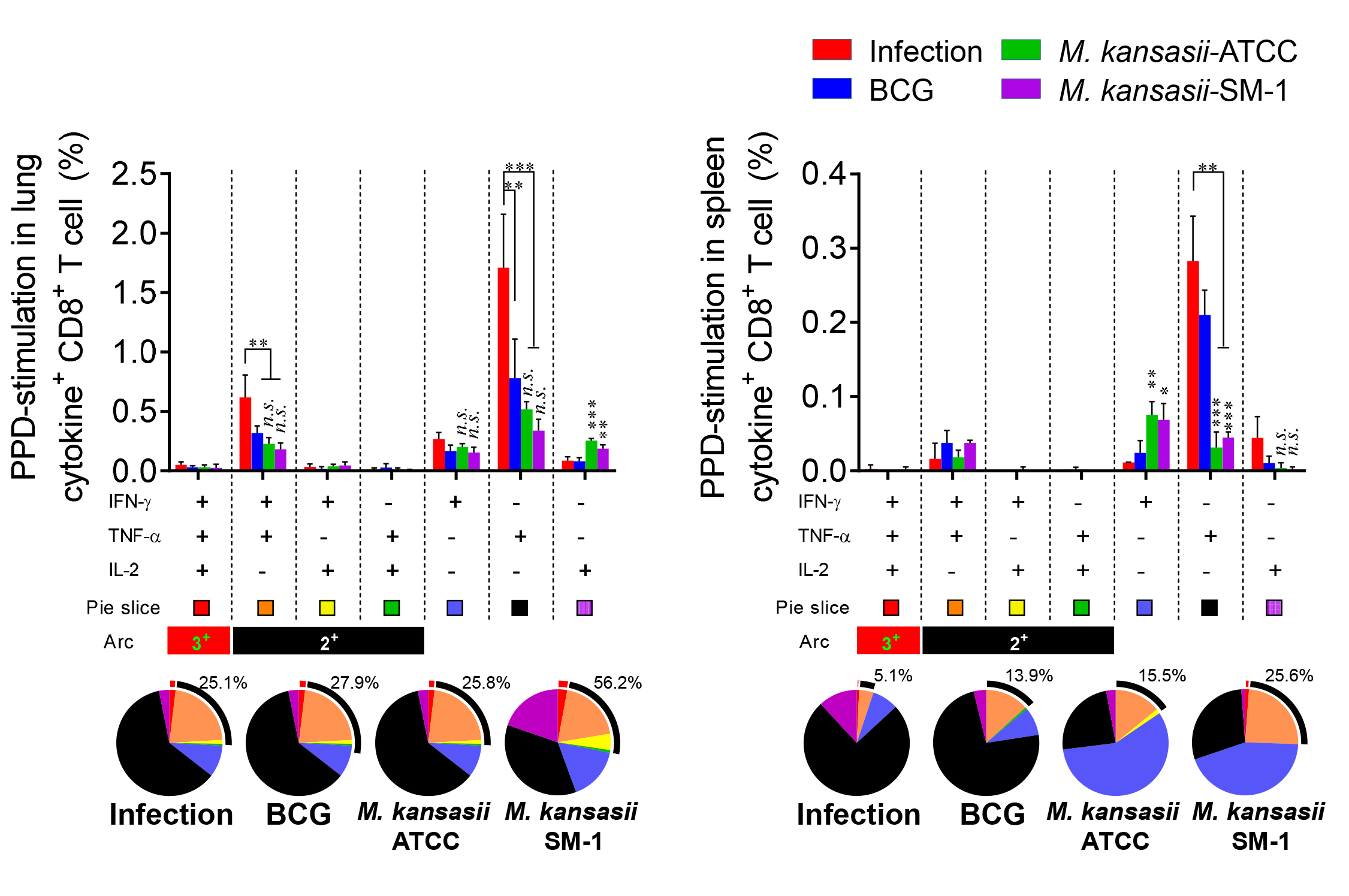
**

**Supplementary Figure 5. Mtb antigen-specific CD8^+^ multifunctional T cell responses in *M. kansasii-*ATCC and *M. kansasii-*SM-1-immunized mice on Mtb H37Rv infection**

Six weeks after the H37Rv challenge, the mice from each group were sacrificed, and their lung and spleen cells were stimulated ex vivo with PPD (5 μg/ml) for 12 hours at 37°C. The percentages of PPD-specific CD8^+^CD62L^lo^CD44^hi^ T cells producing IFN-γ, TNF-α, and/or IL-2 among the cells isolated from the lungs and spleen of each group of mice were analyzed via flow cytometry. The frequencies and proportion of cells coexpressing IFN-γ, TNF-α, and/or IL-2 were represented in bar graph and pie charts. The arc around the pie chart indicates the proportion of T cells simultaneously producing more than two cytokines. The data are presented as the means ± SDs from five mice in each group. One-way ANOVA was used to determine the significance of the differences. **p* < 0.033, ** *p <* 0.002, *** *p <* 0.001; *n.s.*, not significant.
